# Errors in pressure measurements due to changes in pressure transducer levels during adult cardiac surgery: a prospective observational study

**DOI:** 10.1186/s12871-023-01968-7

**Published:** 2023-01-07

**Authors:** Chahyun Oh, Soomin Lee, Seungbin Jeon, Hanmi Park, Woosuk Chung, Man-Shik Shim, Seok-Hwa Yoon, Yoon-Hee Kim, Sun Yeul Lee, Boohwi Hong

**Affiliations:** 1 Department of Anesthesiology and Pain Medicine, Chungnam National University Hospital, Daejeon, Korea; 2 Department of Anesthesiology and Pain Medicine, College of Medicine, Chungnam National University, Daejeon, Korea; 3 Department of Thoracic & Cardiovascular Surgery, Chungnam National University Hospital, Daejeon, Korea; 4 Big Data Center, Biomedical Research Institute, Chungnam National University Hospital, Daejeon, Korea

**Keywords:** Transducer, Hemodynamic monitoring, Phlebostatic axis, Arterial pressure, Central venous pressure, Pulmonary artery pressure, Hypotension

## Abstract

**Background:**

Blood pressure measurement is an essential element during intraoperative patient management. However, errors caused by changes in transducer levels can occur during surgery.

**Methods:**

This single center, prospective, observational study enrolled 25 consecutive patients scheduled for elective cardiac surgery with invasive arterial and central venous pressure (CVP) monitoring. Hydrostatic pressures caused by level differences (leveling pressure) between a reference point (on the center of the left biceps brachii muscle) and the transducers (fixed on the right side of the operating table) for arterial and central lines were continuously measured using a leveling transducer. Adjusted pressures were calculated as measured pressure – leveling pressure. Hypotension (mean arterial pressure < 80, <70, and < 60 mmHg), and CVP (< 6, ≥6 and < 15, or ≥ 15 mmHg) and pulmonary artery pressure (PAP, mean > 20 mmHg) levels were determined using unadjusted and adjusted pressures.

**Results:**

Twenty-two patients were included in the analysis. Leveling pressure ≥ 3 mmHg and ≥ 5 mmHg observed at 46.0 and 18.7% of pooled data points, respectively. Determinations of hypotension using unadjusted and adjusted pressures showed disagreements ranging from 3.3 to 9.4% depending on the cutoffs. Disagreements in defined levels of CVP and PAP were observed at 23.0 and 17.2% of the data points, respectively.

**Conclusions:**

The errors in pressure measurement due to changes in transducer level were not trivial and caused variable disagreements in the determination of MAP, CVP, and PAP levels. To prevent distortions in intraoperative hemodynamic management, strategies should be sought to minimize or adjust for these errors in clinical practice.

**Trial registration:**

cris.nih.go.kr (KCT0006510).

**Supplementary Information:**

The online version contains supplementary material available at 10.1186/s12871-023-01968-7.

## Introduction

Measurement of blood pressure is an essential part of intraoperative patient care. Maintenance of blood pressure and other vital signs within an acceptable clinical range is required to maintain adequate organ perfusion. Moreover, intraoperative blood pressure has been shown to contribute to postoperative outcomes [1–4].

Intraoperative blood pressure is frequently monitored in real time by arterial cannulation in patients with limited cardiovascular capacity and in those expected to experience significant intraoperative blood pressure fluctuation. Proper placement of the transducer is necessary to ensure correct measurement of blood pressure. Ideally, the transducer should be placed at an anatomical point close to the level of the right atrium (phlebostatic axis), in the fourth intercostal space at the midaxillary line [5]. Frequently, however, this is unachievable due to surgical exposure of the chest or the use of a surgical drape, especially during cardiac surgery. Therefore, the transducer is frequently placed at an alternative site, such as a side bar fixed on either side of the operating table.

If the transducer is placed at an alternative position, however, an error can occur when the operating table is tilted, as it changes the level gap between the transducer and the right atrium of the heart. These errors may be overcome by manual adjustment of the transducer every time the position of the operating table is altered. These manual adjustments, however, can be cumbersome to perform and may provide an additional source of error [6, 7]. The present study therefore assessed the effect of introducing a leveling transducer, which measures hydrostatic pressure between the heart and the transducer and quantifying the error resulting from the change in level of the transducer on intraoperative blood pressure in adults undergoing cardiac surgery.

## Materials and methods

### Study design and participants

This single center prospective observational study was conducted at Chungnam National University Hospital, from 7 September 2021 to 1 December 2021. The study was approved by Chungnam National University Hospital’s Institutional Review Board (CNUH 2021–07-073) and was registered prior to patient enrollment at cris.nih.go.kr (KCT0006510, 27/08/2021). This study included consecutive patients aged 20 to 84 years scheduled for elective cardiac surgery with invasive arterial and central venous pressure (CVP) monitoring. Written informed consent was obtained from all enrolled participants before surgery. Patients were excluded if informed consent could not be obtained or if the leveling transducer malfunctioned during surgery, as determined by retrospective review of vital records and indicated by a persistent unrealistic value. This study adhered to the applicable STROBE (Strengthening the Reporting of Observational Studies in Epidemiology) guidelines [8].

### Leveling transducer

In addition to the conventional pressure transducer used to monitor blood pressure, a level transducer (TrueWave™ pressure monitoring set, Edwards Lifesciences, Irvine, CA, USA) was used to measure hydrostatic pressure caused by differences in level between a reference point and the transducers for arterial and central lines. The leveling transducer used a 500 mL pressure bag and non-compressible rigid-walled tubing, identical to that used for blood pressure monitoring and filled with 0.9% saline without heparin.

All transducers (i.e., the leveling, arterial, and central line transducers) were mounted onto the same multi-transducer holder and zeroed to ambient pressure. To ensure that the leveling transducer was functional, the free end of the leveling transducer line was placed 10 cm over the transducer unit and the hydrostatic (leveling) pressure displayed on the monitor was checked. Because 10 cmH_2_O is equal to 7.35 mmHg, 7 or 8 mmHg displayed on the monitor was considered acceptable.

**Fig. 1:**
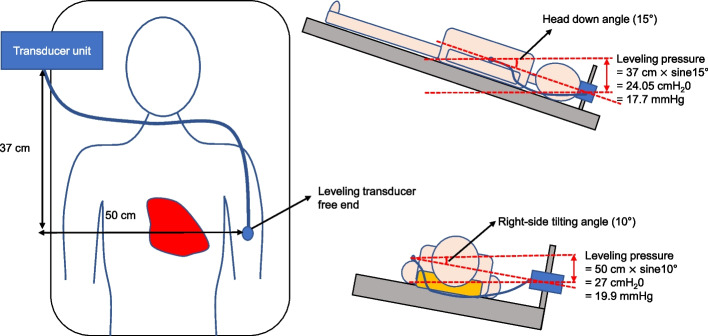
Schematic illustrations of leveling transducer placement and measured leveling pressures in response to changes in position. (Left side) Placements of the transducer unit and its free end. The transducer unit was attached to the rod fixed on the right side of the operating table so that the unit was positioned between the right ear and the vertex of the patient. The distance between the transducer unit and its free end (37 and 50 cm) can vary slightly among individuals. (Right side) Measurements of leveling pressure in the head down (upper panel) and right-side tilting (down panel) positions. The pressures shown are possible values, not actual hydrostatic pressures measured in this study

After confirming that the leveling transducer was functioning properly, the transducer unit was attached to a rod fixed on the right side of the operating table so that the unit was positioned parallel to a line between the right ear and vertex of the patient. The free end of the leveling transducer line was attached to the center of the left upper arm (biceps muscle) of the patient (reference point) (Fig. 1). The level of the transducer unit was adjusted so that the leveling pressure was zero. This process was performed before the placement of the surgical drape, and intraoperative leveling pressure was continuously recorded.

Because the purpose of this study was to quantify errors occurring in usual clinical practice, a blinding method was adopted. To prevent knowledge of leveling pressure introducing alterations in clinical management, the leveling pressure was displayed in the right corner of the monitor screen; after the initial leveling process, this display was covered with non-transparent paper.

### Outcome measures

A mean value of the leveling pressure, automatically processed by an Intellivue MX800 monitor [9] (Philips, Boeblingen, Germany), was used for the entire analysis. The absolute values of the leveling pressures were categorized as < 3 mmHg, ≥3 and < 5 mmHg, ≥5 mmHg.

Adjusted pressure was calculated as measured (unadjusted) pressure – leveling pressure. Hypotension cutoffs were categorized as mean arterial pressure (MAP) < 80 mmHg, < 70 mmHg, and < 60 mmHg [1, 3]. For CVP, mean value automatically processed by the monitor was used. Patients with CVP < 6 mmHg, ≥6 and < 15 mmHg, and ≥ 15 mmHg were categorized as having low, intermediate, and high CVP, respectively [10]. Patients with mean pulmonary artery pressure (mPAP) > 20 mmHg were defined as having high PAP [11].

For example, if measured MAP is 65 mmHg and leveling pressure is 7 mmHg in the right-sided tilted position, then the adjusted MAP would be 58 mmHg. Using a 60 mmHg cut-off for hypotension, the patient would not be hypotensive based on measured MAP but would be hypotensive based on adjusted MAP.

### Data acquisition and processing

All vital data were obtained from the prospective registry of vital signs for surgical patients at Chungnam National University Hospital (CNUH IRB 2019–08-039), which uses a free data collection program (Vital recorder [12] version 1.8, accessed at https://vitaldb.net, Seoul, Republic of Korea).

CVP, PAP, and arterial blood pressure (ABP) were measured using a 7.5 F Swan-Ganz continuous cardiac output thermodilution catheter (CCOmbo V, model 774F75, Edwards Lifesciences LLC) and an arterial catheter. All measured pressures were automatically collected by the Vital recorder at a frequency of 1 Hz. The collected data were extracted at a frequency of 1 Hz and filtered so that measured MAP was > 20 and < 140 mmHg; the measured systolic arterial pressure was < 230 mmHg; the leveling pressure was > − 20 mmHg and < 20 mmHg; the adjusted CVP was > 0 mmHg and < 35 mmHg, and the adjusted mPAP was > 0 mmHg and < 60 mmHg. Absolute leveling pressure < 2 mmHg was considered clinically irrelevant noise and set at 0 mmHg. Additionally, periods with pulse pressure (systolic – diastolic arterial pressure) < 10 mmHg were excluded in determinations of CVP and PAP in order to exclude periods of cardiopulmonary bypass in considerations of the clinical relevance and errors caused by cardiac manipulations. After these filtrations, the means of these values were calculated at 10-second intervals and rounded to integers.

### Post-hoc simulation test

As the reference point used in this study was not the true phlebostactic axis, overestimation of the absolute value of leveling pressure was inevitable when the table was tilted to the left or right side. To evaluate the magnitude of this overestimation and estimate its impact on the results, a set of simulation tests was performed. In the first simulation test, it was assumed that there had been only lateral tilting movements of the table during the study period. Therefore, every occurring leveling pressure was assumed to be overestimated to the proportion of the lateral distance from the reference point to the right atrium (assumed as 20 cm) compared with the lateral distance from the reference point to the transducer unit (50 cm). In this logic, the true leveling pressure was calculated as 0.6 × measured leveling pressure (0.6 derived from [50–20]/50). In the second simulation test, it was assumed that there had been various combinations (lateral tilting and head down or up) of table movements. Therefore, the degree of overestimation should be within the range of 0 to 0.4 (20/50). Thus, the true leveling pressure was calculated as (random value between 0.6 to 1 for each data point) × measured leveling pressure.

### Statistical analysis

Sample size was determined based on an assumption that at least 20 patients are required for essential descriptive statistics. Considering a potential dropout and data loss, we included 25 patients. Analyses were performed both on cohort (pooled) and individual datasets. Disagreements between measured and adjusted values were quantified as time (minutes) and rate (number of conflicting data points/total number of data points). Continuous variables were reported as the mean ± standard deviation (SD) or median (interquartile range [IQR]), depending on the results of Shapiro–Wilk or Kolmogorov-Smirnov tests. All statistical analyses were performed using R software version 4.0.3 (R Project for Statistical Computing, Vienna, Austria).

## Results

**Fig. 2:**
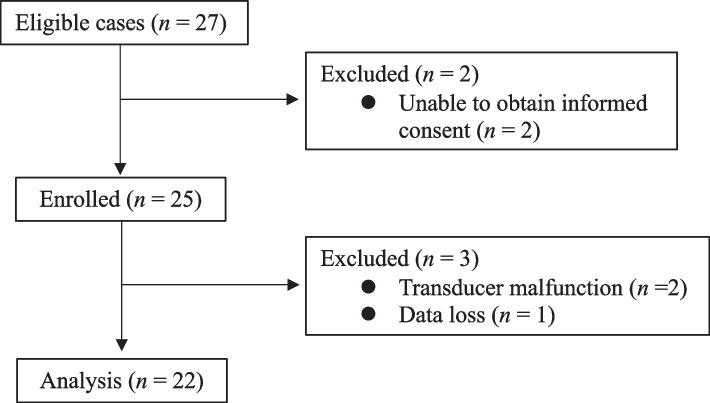
Patient flow diagram

Of the 27 patients eligible for inclusion, two were excluded because informed consent could not be obtained. In addition, two were excluded due to leveling transducer malfunction and one due to loss of data. Thus, 22 patients were analyzed in this study (Fig. 2); their clinical characteristics are shown in Table 1. ABP, CVP, and PAP were recorded for a cumulative 4411.3 min (73.5 h), 3133.5 min (52.2 h), and 3182.0 min (53.0 h), respectively. Adjusted intraoperative MAP, CVP, and mPAP were 73.0 (65.0, 82.0), 12.0 (10.0, 14.0), and 20.0 (16.0, 25.0) mmHg, respectively.

**Table 1:** Patient characteristics

Characteristics	Value
Sex (F/M)	7/ 15 (31.8/ 68.2)
Age (yr)	63.0 (58.0, 69.0)
Height (cm)	161.2 (154.2, 167.8)
Weight (kg)	63.3 (60.8, 77.8)
Surgery type	
● CABG	12 (54.5)
● Valve	7 (31.8)
● Others	3 (13.6)

Data are reported as median (IQR) or number (%). CABG: coronary artery bypass graft surgery

**Fig. 3:**
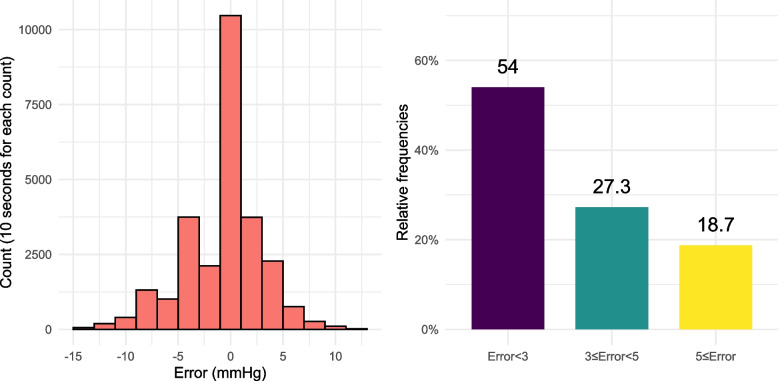
Histogram and bar chart showing the quantity of error in pressure measurement caused by intraoperative level change of the transducer. (Left side) Histogram showing error values (i.e. leveling pressure). (Right side) Bar chart showing the percentages of absolute error values

The distribution of leveling pressure in the pooled dataset is shown in Fig. 3. Of the data points recorded, 46.0% showed leveling pressures ≥3 mmHg, and 18.7% showed ≥5 mmHg, indicative of significant error. Disagreements in the determinations of hypotension between measured and adjusted values in the cohort are summarized in Table 2. Determinations of hypotension showed disagreements ranging from 3.3 to 9.4% depending on the cutoffs. Disagreements in defined levels of CVP and PAP were observed at 23.0 and 17.2% of the measured data points, respectively.

**Table 2:** Differences between measured and adjusted pressures in the patient cohort

Items	Total disagreements, minute (%)	False negative, minute (%)	False positive, minute (%)
ABP (hypotension)			
● MAP < 80 mmHg	277.0 (6.3)	98.3 (2.2)	178.7 (4.1)
● MAP < 70 mmHg	415.3 (9.4)	145.0 (3.3)	270.3 (6.1)
● MAP < 60 mmHg	147.0 (3.3)	83.5 (1.9)	63.5 (1.4)
CVP (low, intermediate, high)	721.7 (23.0)	NA	NA
PAP (mean > 20 mmHg)	546.5 (17.2)	294.7 (9.3)	251.8 (7.9)

*ABP* Arterial blood pressure, *MAP* Mean arterial pressure, *CVP* Central venous pressure, *PAP* Pulmonary artery pressure, *NA* Not available. CVPs < 6 mmHg; ≥6 and < 15 mmHg; and ≥ 15 mmHg were defined as low, intermediate, and high, respectively. False negative indicates the time point with falsely determined as ‘no hypotension’ (or ‘no pulmonary hypertension’) based on an un-adjusted measurement. False positive indicates that time points with falsely determined as hypotension (or pulmonary hypertension) based on an un-adjusted measurement. Note that the % values of the false negative and positive are not the same as the false negative or positive rate (1 – sensitivity or specificity)

The results from the individual data analysis are summarized in Table 3. Leveling pressure ≥ 3 and < 5 mmHg and ≥ 5 were observed at 26.8 ± 17.1% and 13.5 (2.9, 33.9)%, respectively. The determinations of hypotension between measured and adjusted values showed disagreements ranging from median 1.7 to 8.4% depending on the cutoffs. Disagreements in defined levels of CVP and PAP were observed at 22.3% (mean) and 13.4% (median) of the measured data points, respectively.

**Table 3:** Differences between measured and adjusted pressures in individual patients

Items	Duration, minute	Rate, %
Error < 3 mmHg	97.0 ± 46.6	54.6 ± 22.3
Error ≥ 3 and < 5 mmHg	47.2 ± 31.9	26.8 ± 17.1
Error ≥ 5	23.8 (4.5, 45.2)	13.5 (2.9, 33.9)
ABP (hypotension)		
● MAP < 80 mmHg		
▪ Total disagreements	9.4 (5.5, 16.7)	5.4 (3.4, 9.4)
▪ False negative	3.4 (1.2, 6.3)	1.8 (0.6, 4.0)
▪ False positive	5.3 (0.5, 12.7)	3.0 (0.3, 6.8)
● MAP < 70 mmHg		
▪ Total disagreements	12.8 (3.5, 28.0)	8.4 (1.8, 11.9)
▪ False negative	3.1 (0.2, 7.8)	1.5 (0.1, 5.3)
▪ False positive	5.5 (0.2, 18.7)	2.8 (0.1, 8.2)
● MAP < 60 mmHg		
▪ Total disagreements	2.5 (0.0, 7.0)	1.7 (0.0, 4.6)
▪ False negative	0.8 (0.0, 2.5)	0.5 (0.0, 1.6)
▪ False positive	0.2 (0.0, 3.2)	0.1 (0.0, 1.9)
CVP (low, intermediate, high; total disagreements)	24.9 (10.5, 46.0)	22.3 ± 14.7
PAP (mean > 20 mmHg)		
▪ Total disagreements	12.6 (3.5, 38.8)	13.4 (3.3, 26.2)
▪ False negative	1.0 (0.0, 11.8)	0.8 (0.0, 7.7)
▪ False positive	5.9 (0.7, 17.7)	4.2 (0.3, 14.2)

Values are mean ± SD or median (IQR). *ABP* Arterial blood pressure, *MAP* Mean arterial pressure, *CVP* Central venous pressure, *PAP* Pulmonary artery pressure. CVPs < 6 mmHg; ≥6 and < 15 mmHg; and ≥ 15 mmHg were defined as low, intermediate, and high, respectively. False negative indicates the time point with falsely determined as ‘no hypotension’ (or ‘no pulmonary hypertension’) based on an un-adjusted measurement. False positive indicates that time points with falsely determined as hypotension (or pulmonary hypertension) based on an un-adjusted measurement. Note that the % values of the false negative and positive are not the same as the false negative or positive rate (1 – sensitivity or specificity)

The results from the post-hoc simulation tests are summarized in Supplementary material 1. Simulation with only lateral movements of the table showed 18.7 and 3.4% of leveling pressures ≥3 mmHg and ≥ 5 mmHg, respectively. Simulation with various movements showed 31.9 and 10.6% of leveling pressures ≥3 mmHg and ≥ 5 mmHg, respectively.

## Discussion

This study quantified the relative percentage of errors during invasive measurements of blood pressure, including MAP, CVP, and PAP, resulting from changes in transducer level during cardiac surgery in adults. Errors of ≥3 mmHg and ≥ 5 mmHg occurred at 46 and 18.7% of intraoperative data points overall. These errors resulted in errors in the determinations of hypotension (3.3 to 9.4% of disagreements), and CVP and PAP levels (23.0 and 17.2%, respectively). The determinations of CVP and PAP levels were more vulnerable to the leveling pressure as their clinical ranges are narrower than that of MAP. Because clinical management may depend on these determinations, these errors can significantly distort intraoperative hemodynamic management.

Transducer leveling has been reported to be a significant source of clinical error in previous studies [6, 7, 13]. Those studies, which mainly focused on variations in single measurements of CVP, showed significant variations in transducer placement among health care providers. In contrast, the present study mainly focused on deviations of the transducer from the phlebostatic axis caused by positional changes during cardiac surgery. Because this deviation, called leveling pressure, frequently changes and varies among patients, it was continuously monitored using a separate transducer and a high-resolution recording system. As this method adopts a constant reference point (left upper arm), it is free from errors that could have been induced by manual adjustments. The overall error could therefore be quantified, and clinical errors resulting from errors caused by positional changes could be determined. At the same time, this magnitude of error can be thought as the amount of error in the pressure measurement that could be reduced by adopting a leveling transducer into the clinical practice.

Based on current understanding of perioperative outcomes associated with intraoperative hypotension, the percent error determined in this study can be considered significant. Intraoperative hypotension has been associated with detrimental postoperative outcomes [2, 4, 14, 15]. Because even a brief period of hypotension may be harmful [15], the brief misclassifications of hypotension observed in this study may be clinically meaningful, as they may have led to over- or undertreatment.

Although absolute CVP shows little ability to predict fluid-responsiveness [16], it may be clinically useful as a categorical parameter (i.e. low, intermediate, or high) or as a surrogate indicator of right heart congestion [17]. Considering clinical relevancy, the cutoffs for the CVP values were set based on a grey zone approach suggested in a previous study [10]. These values represent sensitivity or specificity of 90% for fluid responsiveness. Thus, the finding of disagreements in CVP levels in the present study deserves clinical attention.

The transducer should be optimally positioned to achieve several goals. First, the transducer should follow changes in the position of the heart during surgery resulting from the frequent manipulation of the operating table to achieve maximal surgical exposure or as a surgical maneuver. Second, the optimal level of the transducer should be verified regularly during surgery, as maintaining the transducer at an accurate and consistent position is paramount. Third, the transducer should be secured from unintentional disturbances. Fourth, because frequent intraoperative arterial blood sampling is required, especially during cardiac surgery, the transducer should be positioned for easy manipulation. Achieving these goals simultaneously during intraoperative period is challenging, especially in cardiac surgery. However, these goals may be accomplished by monitoring leveling pressure and adjusting hemodynamic parameters accordingly.

Another issue associated with the optimal position of the transducer is the relationship between the transducer unit and the reference point. In the present study, the transducer unit was fixed at the right side of the operating table while the reference point was set at the left side of the patient. The transducer unit was positioned on the right side because the organization of the monitoring system in the operating theater at our institution favors a right-sided alignment. The reference point was positioned on the left side to reflect the midaxillary line. This arrangement inevitably results in overestimation of the absolute value of leveling pressure when the table was tilted to the left or right side. This overestimation was indirectly evaluated in the post-hoc simulation tests. These tests were designed a posteriori to reveal the amount of the possibly overrated clinical impact of leveling pressure (simulation 1) and to derive a reasonable estimate (simulation 2). The second simulation test showed non-negligible errors, although the degree was smaller than that of the main result.

This study had several limitations. First, clinical outcomes such as organ injury were not assessed, preventing a determination of the clinical effect of the quantified error. Second, despite operator blinding, an intervention bias may have occurred if a clinician focused on the deviation of the transducer more than usual. Although the leveling pressure could not have been affected, this focus on the transducer may have resulted in tighter hemodynamic management. For example, a clinician might have more actively administered vasoactive agents than usual when the blood pressure was near the cutoff. Third, although the error due to positional change was quantified by measuring leveling pressure, positional change itself, which can also be useful, was not assessed specifically. Owing to this omission, other factors that might have affected the leveling pressure could not be excluded. Fourth, the phlebostatic axis used in this study, the center of the left biceps, is not the true position of the right atrium.

## Conclusion

The errors in pressure measurement due to changes in transducer level were not trivial and caused variable disagreements in the determination of MAP, CVP, and PAP levels. To prevent distortions in intraoperative hemodynamic management, strategies should be sought to minimize or adjust for these errors in clinical practice.

## Supplementary Information

**Additional file 1.**

## Data Availability

The datasets used and/or analysed during the current study are available from the corresponding author on reasonable request.

## Abbreviations

CVP: Central venous pressure
mPAP: Mean pulmonary artery pressure
PAP: Pulmonary artery pressure
MAP: Mean arterial pressure
ABP: Arterial blood pressure
SD: Standard deviation
IQR: Interquartile range

## References

[1] Wesselink EM, Kappen TH, Torn HM, Slooter AJC, van Klei WA (2018). Intraoperative hypotension and the risk of postoperative adverse outcomes: a systematic review. Br J Anaesth.

[2] Sun LY, Wijeysundera DN, Tait GA, Beattie WS (2015). Association of intraoperative hypotension with acute kidney injury after elective noncardiac surgery. Anesthesiology.

[3] Sessler DI, Bloomstone JA, Aronson S, Berry C, Gan TJ, Kellum JA, Plumb J, Mythen MG, Grocott MPW, Edwards MR (2019). Perioperative quality initiative consensus statement on intraoperative blood pressure, risk and outcomes for elective surgery. Br J Anaesth.

[4] Gregory A, Stapelfeldt WH, Khanna AK, Smischney NJ, Boero IJ, Chen Q, Stevens M, Shaw AD (2021). Intraoperative hypotension is associated with adverse clinical outcomes after noncardiac surgery. Anesth Analg.

[5] Saugel B, Kouz K, Meidert AS, Schulte-Uentrop L, Romagnoli S (2020). How to measure blood pressure using an arterial catheter: a systematic 5-step approach. Crit Care.

[6] Sjödin C, Sondergaard S, Johansson L (2019). Variability in alignment of central venous pressure transducer to physiologic reference point in the intensive care unit-a descriptive and correlational study. Aust Crit Care.

[7] Figg KK, Nemergut EC (2009). Error in central venous pressure measurement. Anesth Analg.

[8] von Elm E, Altman DG, Egger M, Pocock SJ, Gøtzsche PC, Vandenbroucke JP (2007). The strengthening the reporting of observational studies in epidemiology (STROBE) statement: guidelines for reporting observational studies. Lancet.

[9] Roger C, Muller L, Riou B, Molinari N, Louart B, Kerbrat H, Teboul JL, Lefrant JY (2017). Comparison of different techniques of central venous pressure measurement in mechanically ventilated critically ill patients. Br J Anaesth.

[10] Biais M, Ehrmann S, Mari A, Conte B, Mahjoub Y, Desebbe O, Pottecher J, Lakhal K, Benzekri-Lefevre D, Molinari N (2014). Clinical relevance of pulse pressure variations for predicting fluid responsiveness in mechanically ventilated intensive care unit patients: the grey zone approach. Crit Care.

[11] Humbert M, Kovacs G, Hoeper MM, Badagliacca R, Berger RMF, Brida M, Carlsen J, Coats AJS, Escribano-Subias P, Ferrari P (2022). 2022 ESC/ERS guidelines for the diagnosis and treatment of pulmonary hypertension. Eur Heart J.

[12] Lee HC, Jung CW (2018). Vital recorder-a free research tool for automatic recording of high-resolution time-synchronised physiological data from multiple anaesthesia devices. Sci Rep.

[13] Avellan S, Uhr I, McKelvey D, Sondergaard S (2017). Identifying the position of the right atrium to align pressure transducer for CVP. J Clin Monit Comput.

[14] Wachtendorf LJ, Azimaraghi O, Santer P, Linhardt FC, Blank M, Suleiman A, et al. Association Between Intraoperative Arterial Hypotension and Postoperative Delirium After Noncardiac Surgery: A Retrospective Multicenter Cohort Study. Anesth Analg. 2022;134(4):822–33. 10.1213/ANE.0000000000005739 34517389

[15] Walsh M, Devereaux PJ, Garg AX, Kurz A, Turan A, Rodseth RN, Cywinski J, Thabane L, Sessler DI (2013). Relationship between intraoperative mean arterial pressure and clinical outcomes after noncardiac surgery: toward an empirical definition of hypotension. Anesthesiology.

[16] Marik PE, Baram M, Vahid B (2008). Does central venous pressure predict fluid responsiveness? A systematic review of the literature and the tale of seven mares. Chest.

[17] De Backer D, Vincent JL (2018). Should we measure the central venous pressure to guide fluid management? Ten answers to 10 questions. Crit Care.

