# Supplementary material for: Errors in pressure measurements due to changes in pressure transducer levels during adult cardiac surgery: a prospective observational study
Source: BMC Anesthesiol. 2023 Jan 7;23:8. doi: 10.1186/s12871-023-01968-7 (PMC9824971; doi:10.1186/s12871-023-01968-7)
Supplement: Supplementary file 1 — Additional file 1. [file 12871_2023_1968_MOESM1_ESM.docx]

**Supplementary material 1: post-hoc simulation test**

As the reference point used in this study was not the true phlebostactic axis, overestimation of the absolute value of leveling pressure was inevitable when the table was tilted to the left or right side. To estimate the magnitude of this overestimation and evaluate its impact on the pressure measurements, a set of simulation tests was performed.

**1. Simulation 1: with only lateral tilting movements**

It was assumed that there had been only lateral tilting movements of table during the study period. Therefore, every occurring leveling pressure was assumed to be overestimated to the proportion of the lateral distance from the reference point to right atrium (assumed as 20 cm) compared with the lateral distance from the reference point to the transducer unit (50 cm). In this logic, the true leveling pressure was calculated as 0.6 $\times$ measured leveling pressure (0.6 derived from [50-20]/50)


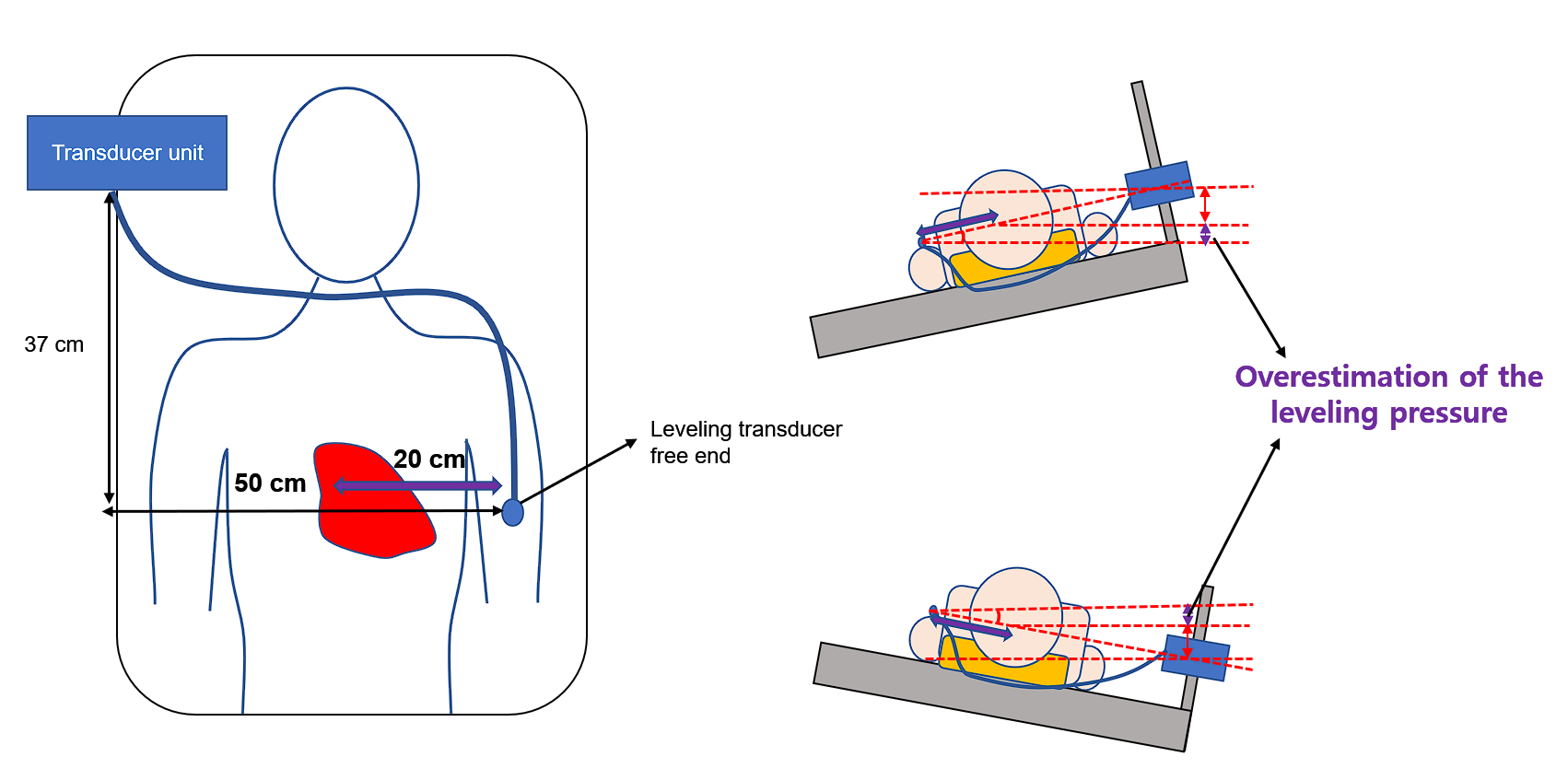


**2. Simulation 2: with various movements**

It was assumed that there had been various combinations (lateral tilting and head down or up) of table movements. Therefore, the degree of overestimation should be within the range of 0 to 0.4 (20/50). Thus, the true leveling pressure was calculated as (random value between 0.6 to 1 for each data point) $\times$ measured leveling pressure.

**3. Results**


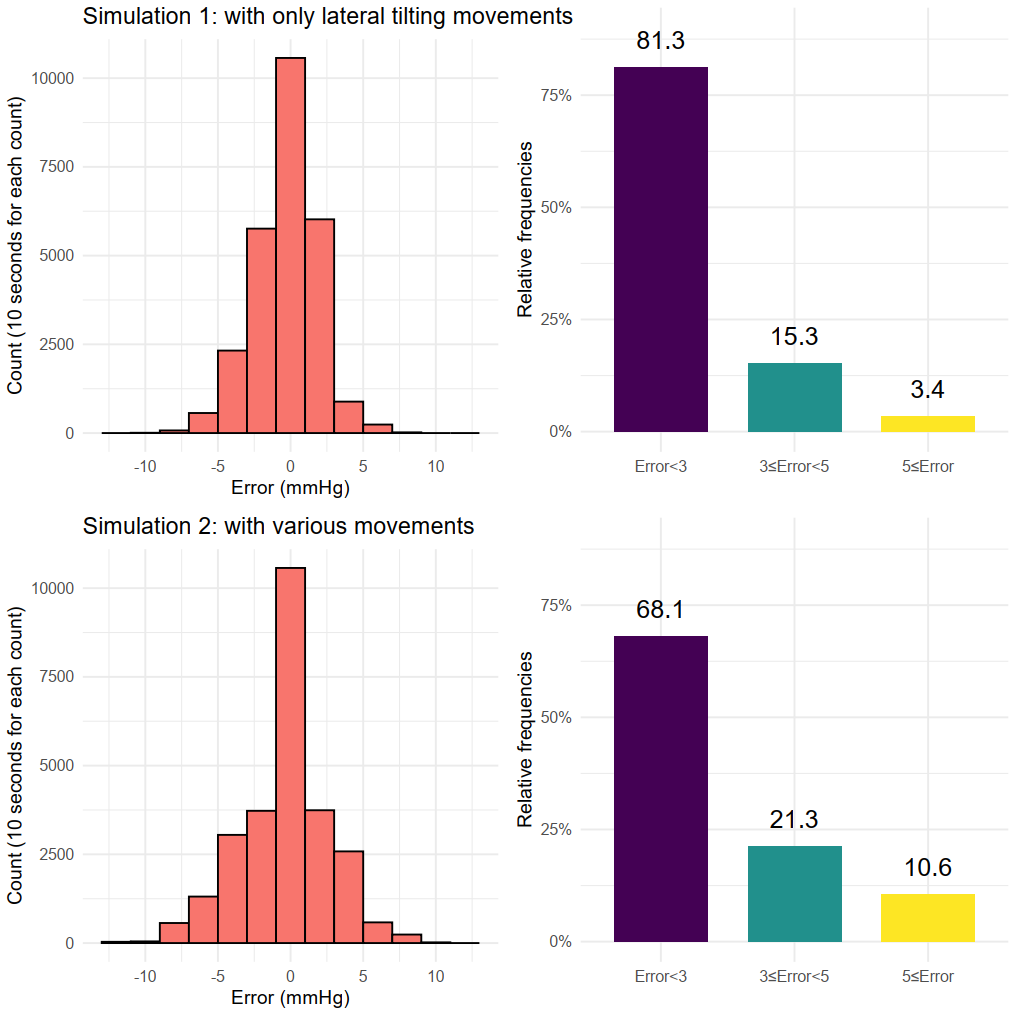


Histogram and bar chart showing the quantity of error in pressure measurement caused by intraoperative level change of the transducer.

| **Simulation 1: with only lateral tilting movements** | | | |
| --- | --- | --- | --- |
| Items | Total disagreements,  minute (%) | False negative,  minute (%) | False positive,  minute (%) |
| ABP (hypotension) |  |  |  |
| - MAP <80 mmHg | 143.3 (3.2) | 70.5 (1.6) | 72.8 (1.7) |
| - MAP <70 mmHg | 271.5 (6.2) | 109.7 (2.5) | 161.8 (3.7) |
| - MAP <60 mmHg | 98.0 (2.2) | 56.7 (1.3) | 41.3 (0.9) |
| CVP (low, intermediate, high) | 486.8 (15.5) | NA | NA |
| PAP (mean >20 mmHg) | 315.0 (9.9) | 244.5 (7.7) | 70.5 (2.2) |
| **Simulation 2: with various movements** | | | |
| Items | Total disagreements,  minute (%) | False negative,  minute (%) | False positive,  minute (%) |
| ABP (hypotension) |  |  |  |
| - MAP <80 mmHg | 194.2 (4.4) | 91.5 (2.1) | 102.7 (2.3) |
| - MAP <70 mmHg | 345.7 (7.8) | 136.0 (3.1) | 209.7 (4.8) |
| - MAP <60 mmHg | 126.2 (2.9) | 76.7 (1.7) | 49.5 (1.1) |
| CVP (low, intermediate, high) | 547.8 (17.5) | NA | NA |
| PAP (mean >20 mmHg) | 438.2 (13.8) | 294.5 (9.3) | 143.7 (4.5) |

ABP: arterial blood pressure, MAP: mean arterial pressure, CVP: central venous pressure, PAP: pulmonary artery pressure, NA: not available. CVPs <6 mmHg; ≥6 and <15 mmHg; and ≥15 mmHg were defined as low, intermediate, and high, respectively. False negative indicates the time point with falsely determined as ‘no hypotension’ (or ‘no pulmonary hypertension’) based on an un-adjusted measurement. False positive indicates that time points with falsely determined as hypotension (or pulmonary hypertension) based on an un-adjusted measurement. Note that the % values of the false negative and positive are not the same as the false negative or positive rate (1 – sensitivity or specificity)
